# Clarity on the Type of Review. Comment on “Value Cocreation in Health Care: Systematic Review”

**DOI:** 10.2196/38457

**Published:** 2022-07-11

**Authors:** Fnu Kajal

**Affiliations:** 1 University of Arizona Tucson, AZ United States

**Keywords:** value cocreation, health care, patient value, health care professional value, systematic review

I have read the systematic review titled, “Value Cocreation in Health Care: Systematic Review,” by Peng et al [1]. The objective of the paper was to identify and review the literature as the area of value cocreation is new to health care. The topic is very relevant as there is a need to add value to health care that will ultimately help to reduce health inequities.

While this review summarizes the literature well, it does not qualify as a systematic review. Foremost is the lack of a clear question the review seeks to answer. A systematic review is usually conducted to answer a question; in this case, the authors seem to have conducted a scoping or narrative review systematically.

The authors themselves state that this area of research is new and the literature is fragmented. Thus, it would have been better to have conducted a scoping review rather than a systematic review [2]. Further, the search terms for this review do not seem to be adequate to capture all research on the subject. For example, the phrases used in the search strategy do not include “respectful care,” which is often used in value cocreation in health care systems.

In addition, a high-quality systematic review follows PRISMA (Preferred Reporting Items for Systematic Reviews and Meta-Analyses) guidelines and checklists. This must be addressed in reference to PICO (population, intervention, comparators, and outcome). The systematic review lacks clarity on comparators and does not provide a list of all outcomes for which data were sought [3]. The MMAT (Mixed Method Appraisal Tool) does mention the quality of studies but lacks the anticipated risk of bias assessment in individual studies. Further, the authors have also not detailed any variability between the studies through heterogeneity, which might have impacted the interpretation of the results [4].

Most problematic, however, is the framework developed and presented in this review. The methodology of mapping the findings onto an existing theory is not a standard method. The authors need to justify why this method was adopted. The utility of this framework, therefore, is also not clear.

This area of research is clearly very relevant, and the authors have tried to put together the literature on this, but their systematic review needs more details at the granular level for a better understanding of the gaps and solutions to address areas of concern in the future.

## Abbreviations

MMAT: Mixed Method Appraisal Tool
PICO: population, intervention, comparators, and outcome
PRISMA: Preferred Reporting Items for Systematic Reviews and Meta-Analyses

## References

[1] Peng Y, Wu Tailai, Chen Zhuo, Deng Zhaohua (2022). Value cocreation in health care: Systematic review. J Med Internet Res.

[2] Peters M, Godfrey Christina M, Khalil Hanan, McInerney Patricia, Parker Deborah, Soares Cassia Baldini (2015). Guidance for conducting systematic scoping reviews. Int J Evid Based Healthc.

[3] Page M, McKenzie Joanne E, Bossuyt Patrick M, Boutron Isabelle, Hoffmann Tammy C, Mulrow Cynthia D, Shamseer Larissa, Tetzlaff Jennifer M, Akl Elie A, Brennan Sue E, Chou Roger, Glanville Julie, Grimshaw Jeremy M, Hróbjartsson Asbjørn, Lalu Manoj M, Li Tianjing, Loder Elizabeth W, Mayo-Wilson Evan, McDonald Steve, McGuinness Luke A, Stewart Lesley A, Thomas James, Tricco Andrea C, Welch Vivian A, Whiting Penny, Moher David (2021). The PRISMA 2020 statement: an updated guideline for reporting systematic reviews. BMJ.

[4] Higgins J, Thompson Simon G (2002). Quantifying heterogeneity in a meta-analysis. Stat Med.

